# High-Specificity and Sensitivity Imaging of Neutral Lipids Using Salt-Enhanced MALDI TIMS

**DOI:** 10.1021/jasms.5c00202

**Published:** 2025-09-04

**Authors:** Kameron R. Molloy, Martin Dufresne, Madeline E. Colley, Lukasz G. Migas, Raf Van de Plas, Jeffrey M. Spraggins

**Affiliations:** † Department of Chemistry, 5718 Vanderbilt University, Nashville, Tennessee 37235, United States; ‡ Mass Spectrometry Research Center, Vanderbilt University, Nashville, Tennessee 37235, United States; § Department of Cell and Developmental Biology, Vanderbilt University, Nashville, Tennessee 37232, United States; ∥ Delft Center for Systems and Control, 2860 Delft University of Technology, 2628 Delft, Netherlands; ⊥ Department of Biochemistry, Vanderbilt University, Nashville, Tennessee 37205, United States; # Department of Pathology, Microbiology and Immunology, Vanderbilt University Medical Center, Nashville, Tennessee 37205, United States

## Abstract

Neutral lipids are vital to various cellular processes and disease pathologies. However, their characterization by matrix-assisted laser desorption/ionization imaging mass spectrometry (MALDI IMS) remains challenging due to poor ionization efficiency and difficulties distinguishing subtle structural differences among numerous isomeric and isobaric species. In this study, we enhanced neutral lipid detection by incorporating isotonic metal–cation washes into our MALDI IMS sample preparation workflow. Resulting salt adducts improved neutral lipid isobar and isomer separation by using trapped ion mobility spectrometry (TIMS). This approach increased both sensitivity and specificity for neutral lipid IMS experiments across multiple organ types, including murine brain, rabbit adrenal gland, human colon, and human kidney. Comparative analyses revealed that the most effective salt wash was tissue-dependent. However, the Na^+^ carbonate buffer solution (CBS) wash showed the greatest overall increase in neutral lipid detection. These findings provide a robust framework for mapping neutral lipids across multiple tissues and disease states and allow for the detailed characterization of neutral lipid isomers and isobars in complex biological tissues.

## Graphical abstract

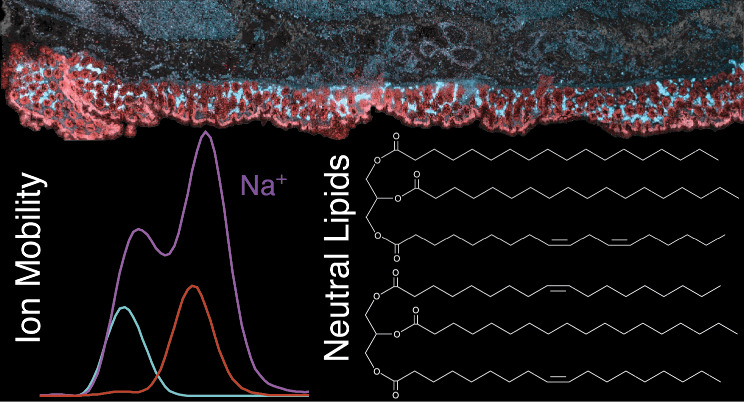

## Introduction

Neutral lipids are a diverse group of biomolecules that include sterols (ST) such as cholesterol, cholesteryl esters (CE) and steroids, ceramides (Cer), hexosylceramides (HexCer), fatty acyl esters of hydroxy fatty acids (FAHFA), mono-, di-, triacylglycerols (MAG, DAG, and TAG), and others.^1^ These lipids play crucial roles in cell signaling, energy storage, membrane dynamics, and maintaining lipid homeostasis in biological systems.^2–4^ For instance, DAGs are necessary for activating intracellular proteins such as protein kinase C in the brain, and cholesterol modulates cellular membrane fluidity and is associated with various diseases.^5,6^ Furthermore, neutral lipids have been implicated in neurodegenerative disorders such as Alzheimer’s disease, as shown by Akyol et al., who reported the correlation between Cer and HexCer levels and disease severity.^7^ While lipidomic analyses of tissue homogenates and biofluids have highlighted the importance of neutral lipids both in healthy and disease conditions, relatively few studies have explored the spatial distribution of these molecular species *in situ*.^2,7–9^ As lipids encompass a wide variety of structural diversity, there is a clear need for highly sensitive techniques that provide precise structural characterization and the capability to connect these essential molecules to specific features and cell types.

Matrix-assisted laser desorption/ionization imaging mass spectrometry (MALDI IMS) is a versatile analytical tool that combines the molecular specificity of mass spectrometry with high-resolution tissue mapping. Given its high sensitivity, broad dynamic range, and high spatial resolution mapping capabilities, it is particularly well suited for answering spatially driven biological questions.^8,10–13^ IMS excels at visualizing the relative abundance and localization of a variety of molecules in tissue, including metabolites, lipids, peptides, and proteins.^11,14–17^ This enables histologically relevant spatiomolecular analyses of thin tissue sections, providing a more comprehensive view of biological processes spanning molecular classes and spatial scales. While the spatial distributions of phospholipids, sulfatides, and sphingomyelins are generally well characterized by MALDI IMS, neutral lipids often present a challenge due to their limited ionization efficiency arising from the absence of proton-exchanging functional groups.^11,18,19^

Improving neutral lipid sensitivity for IMS is an ongoing area of interest, with previous studies exploring both sample preparation and instrumental strategies to overcome this challenge in an imaging context.^20–27^ Strategies of improving neutral lipid sensitivity by MALDI IMS include silver sputter coating or applying ionic solutions directly to the sample to promote metal cationization. Of these approaches, using ionic solutions (i.e., salt doping) like Na^+^, K^+^, and Li^+^ chloride,^25,28,29^ Na^+^ citrate,^25^ and carbonate buffer solution^25,26^ as cationization agents has shown particular promise. Other post-ionization approaches, such as MALDI-2, enhance the detection of neutral lipids without requiring additional sample preparation by driving protonation after the initial MALDI desorption event.^30,31^ Though all of these approaches have been effective at enhancing neutral lipid detection, they require multistep sample preparation and advanced instrumentation or are incompatible with subsequent analyses using the same tissue section as part of multimodal studies (e.g., histological staining, immunofluorescence microscopy, spatial transcriptomics, etc.).

Beyond sensitivity enhancements, specificity improvements are key to providing confident annotations and localizations of detected neutral lipids. The structural diversity within neutral lipid subclasses complicates structural identification. For example, isobars (similar mass) and isomers (same exact mass) cannot be easily distinguished by their mass-to-charge ratios (*m*/*z*) alone on many mass spectrometers.^32^ Consequently, IMS images may ambiguously represent multiple isomeric and/or isobaric ions sharing similar *m*/*z* values.^33^ Orthogonal separation techniques, like ion mobility mass spectrometry or derivatization strategies, are capable of distinguishing closely related lipid species.^34,35^ This greatly enhances the number of neutral lipid identifications obtained from a single analysis.

Past research has shown that metal–cation doping of the sample facilitates greater separation of carbohydrates, di-, and tri-saccharide isomers using electrospray ionization with ion mobility mass spectrometry.^36^ More recently, this approach was successfully employed to enhance the separation of phospholipid isomers using silver nitrate as the doping agent.^37^ Although there are many different types of ion mobility, trapped ion mobility spectrometry (TIMS) enables high mobility resolving power separation of target analytes, allowing for the separation of lipid isomers at time scales compatible with imaging experiments.^35^ MALDI TIMS IMS, in combination with metal–cation doping, offers an exciting approach for the in-depth investigation of neutral lipid structural diversity within a spatial context.

Here, we introduce a new MALDI imaging workflow using salt doping and high-resolution TIMS to enhance the sensitivity and specificity of neutral lipids in multiple tissue types. We increased neutral lipid sensitivity in an untargeted fashion by using an isotonic salt wash in place of the conventional ammonium formate wash before matrix sublimation.^38^ Utilizing TIMS and leveraging unique conformations of neutral lipids created by salt adducts, we separated and mapped the distinct spatial distributions of neutral lipid isomers in murine brain, rabbit adrenal gland, human colon, and human kidney tissues.

## Materials and Methods

### Materials

HPLC-grade acetonitrile (ACN), chloroform, methanol, methyl *tert*-butyl ether (MTBE), and tetrahydrofuran (THF) were acquired from Fisher Scientific (Pittsburgh, PA, USA). Glyceride lipid standards were purchased from Cayman Chemical (Ann Arbor, MI, USA), and all other standards were purchased from Avanti Polar Lipids (Alabaster, AL, USA). Detailed lipid standard information is provided in Table 1. Silver nitrate (AgNO_3_), ammonium formate (AF), sodium and potassium carbonate, bicarbonate, and acetate were purchased from Sigma-Aldrich (St. Louis, MO, USA). α-Cyano-4-hydroxycinnamic acid (CHCA) was purchased from Bruker Daltonik (Bremen, Germany), and an aminated cinnamic acid analog matrix^39^ was provided by the Vanderbilt Institute of Chemical Biology Molecular Design and Synthesis Center (Vanderbilt University, Nashville, TN).

**Table 1:** Neutral Lipid Isomer Standard Information

Abbreviation	Formal Name	Acyl Chains	MW	Molecular Formula	Company	Item Number
MAG	1-Arachidonoyl glycerol	20:4	378.277	C_23_H_38_O_4_	Cayman Chemical	62150
MAG	2-Arachidonoyl glycerol	20:4	378.277	C_23_H_38_O_4_	Cayman Chemical	62160
TAG	1,2-Distearoyl-3-linoleoyl-rac-glycerol	18:0/18:0/18:2	886.798	C_57_H_106_O_6_	Cayman Chemical	28179
TAG	1,3-Dioleoyl-2-stearoyl glycerol	18:1/18:0/18:1	886.798	C_57_H_106_O_6_	Cayman Chemical	26889
HexCer	Glucosyl(β) ceramide (d18:1/24:1(15Z))	18:1/24:1	809.674	C_48_H_91_NO_8_	Avanti Research	860549
HexCer	Galactosyl(α) ceramide (d18:1/24:1(15Z))	18:1/24:1	809.674	C_48_H_91_NO_8_	Avanti Research	860432
HexCer(S)	Galactosyl(β) dimethyl sphingosine	18:1	489.367	C_26_H_51_NO_7_	Avanti Research	860579
HexCer(S)	Glucosyl(β) sphingosine	20:1	489.367	C_26_H_51_NO_7_	Avanti Research	860438
Cer	C24 Ceramide	18:1/24:0	649.637	C_42_H_83_NO_3_	Avanti Research	860524
Cer	C24:1 Dihydroceramide	18:0/24:1	649.637	C_42_H_83_NO_3_	Avanti Research	860629

### Sample Preparation

Lipid standards were dissolved in appropriate solvents with a final concentration of 10 mM: TAGs in MTBE, MAGs in ACN, HexCers in methanol, HexCer­(S), and Cer standards in chloroform. CHCA matrix (7 mg/mL) was prepared using a 50:50 ACN:H_2_O mixture. Salt solutions, including sodium carbonate buffer (Na^+^ CBS), potassium carbonate buffer (K^+^ CBS), AgNO_3_, and AF, were made. To account for the multivalent nature of the carbonate salts, Na^+^ CBS and K^+^ CBS were diluted 2.5× to create a nearly isotonic solution. Individual molarities are listed in Table S1. Salt solutions were stored at 4 °C. Na^+^ CBS and K^+^ CBS were comprised of a carbonate buffer solution spiked with acetate as outlined by Dufresne et al.^26,27^

Standards were spotted on an MTP 384 ground steel target plate (Bruker Daltonik, Bremen, Germany). First, CHCA (0.50 μL) was spotted on the target plate. Once dry, the standard solution (0.25 μL) was applied, followed by 0.50 μL of salt solution (omitted AF as a control), then 0.50 μL of CHCA. The layered spot was allowed to dry at room temperature. Triplicate spots of individual standards and mixed isomer pairs were spotted for all standards. When spotting, the salt solutions were diluted 2.5× to promote proper crystallization.

Fresh frozen tissues, including human colon (collected by the Cooperative Human Tissue Network from deidentified, consented donors under Institutional Review Board (IRB)-approved protocol #031078), human kidney (obtained through the Vanderbilt Cooperative Human Tissue Network from a disease-free tumor-associated nephrectomy placed on ice within 2 h of surgery. IRB protocol #181822),^40^ rat brain (BioIVT, Hicksville, NY, USA), and mature rabbit adrenal gland (Pel-Freez Biologicals, Rogers, AR, USA) were cryosectioned on a Leica CM3050 cryostat (Leica Microsystems GmbH, Wetzlar, Germany) at 10 μm thickness, and thaw-mounted onto indium tin oxide coated glass slides (Delts Technology, Loveland, CO, USA). The samples were then submerged in either Na^+^ CBS, K^+^ CBS, AgNO_3_, or AF three times for 45 s each. Tissues were then allowed to dry in a vacuum desiccator for 10 min. Matrix (Vandy37), 1.2 μg/mm^2^, was prepared in THF and applied to all slides using an HTX SubliMATE (HTX Technologies, LLC, Chapel Hill, NC, USA). During sublimation, the coldfinger was cooled using dry ice and acetone. Slides were allowed to cool for 5 min before heating to 250 °C for 10 min at 10 mTorr. Next, dry ice was removed, dropping the temperature to 200 °C before adding a preheated 85 °C hot, metallic puck to the SubliMATE. Once the device had reached room temperature, slides were removed and annealed on a hot plate at 100 °C for 20 s.

### MALDI TIMS IMS

Spotted lipid standards and tissue analyses were performed on a timsTOF fleX MS instrument equipped with a SRIG mobility funnel (Bruker Daltonik, Bremen, Germany). To inform subsequent tissue analyses, we evaluated the isomer separation efficiency by TIMS with lipid standards. These data were acquired with a 20 μm pitch before obtaining MALDI TIMS IMS on tissues including murine brain, rabbit adrenal gland, human colon, and human kidney at a 10 μm pitch. Across all studies (standard and tissue), TIMS data were collected with a collision cell in voltage of 220 V, a source temperature of 50 °C, and a TIMS in pressure of 2.65 ± 0.03 mTorr. All data were collected in positive ion mode, with ramp rates between 0.01–0.18 V/ms and inverse mobility (1/*K*_0_) ranges falling within 0.8–1.8 V·s/cm^2^. A comprehensive table of instrument parameters is provided in Table S1.

### MALDI FT-ICR MS

High resolving power (>400,000 resolving power at *m*/*z* 600) mass spectra of Na^+^ CBS doped human colon tissue were acquired using a 15T MALDI Fourier transform ion cyclotron resonance (FT-ICR) mass spectrometer (Bruker Daltonics, Billerica, MA, USA) equipped with a ParaCell detector. All FT-ICR data were collected at a 4 M file size using 38% laser power. Data for DAG isobars was collected using 20,000 shots with an *m*/*z* range of 253.4–900. Precursor ions were mass-selected using a linear quadrupole (*m*/*z* window: 573.0 ± 1.75 Da) and fragmented by sustained off-resonance irradiation collision-induced dissociation (pulsed argon, 0.25 s, −500 Hz irradiation). MS of PC 36:4 was collected using 1,000 shots with an *m*/*z* range of 506.8–1000. The quadrupole was used to mass select the precursor ion (*m*/*z* window: 855.7 ± 5 Da). Finally, data for TAG 50:1 was collected using 100 shots with an *m*/*z* range of 460.8–1000. No isolation or fragmentation was performed on this species.

### Data Processing and Analysis

All mass spectrometry data and ion mobility images were visualized in Compass DataAnalysis and SCiLS Lab (Bruker Daltonik, Bremen, Germany). Ion mobility images and spectra were generated by using a ± 0.01 Da window. All neutral lipid isomer standard mobility separations are described using two-peak resolution (*R*_pp_), calculated from equations outlined by Dodds et al.^41^ *R*_pp_ for each separation was only reported if the % valley separation was greater than 45%.^42^ These data were also processed in collaboration with the Van de Plas laboratory at TU Delft. Using their in-house designed IMS data processing and annotation software (*e.g.*, *annotine*), the spectra from each imaging run were mass aligned, mass calibrated, normalized, and annotated.^43,44^ Lipidomic profiles of each tissue were generated from LC-MS/MS data collected on a timsTOF Pro2 MS instrument (Bruker Daltonik, Bremen, Germany). Full experimental details are defined in Supplementary Section S1. In *annotine*, the LC-MS/MS libraries were used in conjunction with the structural LIPIDMAPS database to make lipid annotations.^45^ Identifications were made by using a 5 ppm threshold, and ion images were used to qualitatively validate each annotation. Lipid identifications are provided with accompanying mass errors (ppm) based on theoretical values from LIPIDMAPS and our preprocessed measured values.

## Results

### Enhanced Sensitivity and Molecular Coverage

We tested the effect of four salt washes (Na^+^ CBS, K^+^ CBS, AgNO_3_, and AF) on four tissue types: rat brain, rabbit adrenal gland, human colon, and human kidney. These tissues were selected based on their distinct lipid classes, which collectively provided a variety of neutral lipid species. All data were generated in positive ion mode, and the maximum number of neutral lipids detected (i.e., putatively annotated) defined the optimal sample preparation conditions. Comparing the number of annotated molecular species revealed that between the four washes tested, Na^+^ CBS provided the greatest improvement in sensitivity for neutral lipids. Percent increases highlighted in Table 2 show that the Na^+^ CBS wash had the largest improvement in neutral lipids detected compared to a typical AF wash across all tissues.

**Table 2:** Percent Increase (Number of Neutral Lipid Annotations) between Each Wash As Compared to Tissues Washed with Ammonium Formate

Tissue	Na^+^ CBS	K^+^ CBS	AgNO_3_	AF
Adrenal Gland	463% (45)	138% (19)	88% (15)	8
Brain	64% (23)	57% (22)	21% (17)	14
Colon	78% (73)	44% (59)	34% (55)	41
Kidney	400% (15)	100% (6)	267% (11)	3

A complete table of annotations is provided in and average mass spectra for all tissues are available in . TAGs and DAGs contributed the most to the overall increase in the number of annotations (Figure 1a). These species ionized best when tissues were washed with Na^+^ CBS, which aligns with previous studies that observed a sensitivity enhancement when using sodium during sample preparation.^25,27^ The greatest increase in the number of annotated neutral lipid species was found in TAGs, which is consistent with the LC-MS/MS results. Confirmational LC-MS/MS experiments found TAGs to account for over one-third of the neutral lipids detected in colon, kidney, and adrenal tissues (Figure S2).

**Figure 1:**
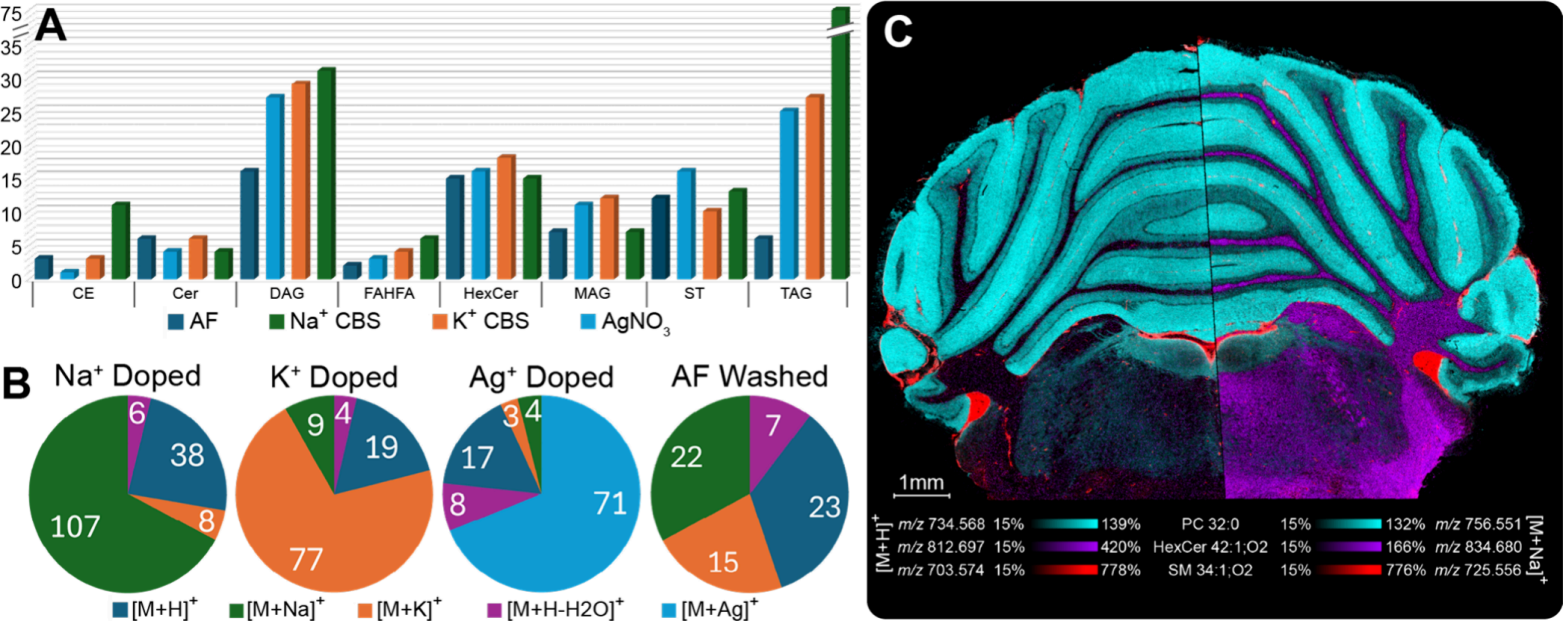
Total number of neutral lipids annotated across all tissues for each salt wash, organized by the major neutral lipid subclasses (A). Frequency of adduct annotations summed across all four organ tissues for each salt wash (B). Two rat cerebellum serial sections washed with AF (left) and Na^+^ CBS (right) were imaged and then processed together using SCiLS Lab (C).

Another factor contributing to the sensitivity enhancement is the ability of salt washes to simplify the adduct profiles of detected neutral lipids. The distribution of adduct types observed for each wash across all tissues is shown in Figure 1b. Salt doping with Na^+^ CBS, K^+^ CBS, and AgNO_3_ washes, improved neutral lipid sensitivity and promoted the formation of the targeted cationic adduct for each wash (Figure 1b). In contrast, the AF wash exhibited much lower sensitivity, likely due to the tendency of neutral lipids to ionize via cationization rather than protonation, leading to their total ion counts being distributed across multiple *m*/*z* values.^25,46,47^ Thus, a notable advantage of salt doping is minimizing nonspecific adduct formation, including non-wash-related metal cationization and protonation.

In traditional MALDI IMS workflows using AF washes, phospholipids and other charged lipids are most commonly ionized as the protonated adduct.^38,48^ Neutral lipids do not preferentially ionize in the protonated form; therefore, including salts in the sample preparation workflow enhances their sensitivity without damaging the tissue or sacrificing the ability to perform downstream multimodal analyses. While some neutral lipids can be detected without salt doping, their signal intensity is greatly diminished compared to a salt-doped sample. This is illustrated in Figure 1c. When a rat cerebellum section was washed with AF, HexCer 42:1;O2 was detected at low abundance as [M + H]^+^ (left). In contrast, when a serial section was washed with Na^+^ CBS, this same lipid was detected as the [M + Na]^+^ adduct with a much higher abundance (right). Advantageously, species besides neutral lipids (e.g., phospholipids and sphingolipids) are also ionized as the metal–cation adduct and show no signs of wash-induced delocalization. For example, PC 32:0 and SM 34:1;O2 maintain their expected localization to gray matter and vascular regions of the cerebellum, respectively (Figure 1c). Overall, salt doping represents a simple, cost-effective, and broadly accessible strategy to enhance the detection of neutral lipids.

### Enhanced Specificity

#### Isomeric Standard Separations

To assess the unique impact a variety of salt adducts have on the geometry and, consequently, the mobility of distinct neutral lipid isomers, we analyzed five neutral lipid isomer pair standards doped with four salt solutions (Na^+^ CBS, K^+^ CBS, AgNO_3_, and AF). Double-bond positional isomers, chain length isomers, regioisomers, and stereoisomers were selected for comparison. A detailed list of standard information is outlined in Table 1, and structural differences in each isomeric type are summarized in Figure S3. The separation of each isomer pair was evaluated with every salt solution. Figure 2 highlights the ion mobility spectrum of the salt adduct that exhibited the most resolved separation for each standard.

**Figure 2:**
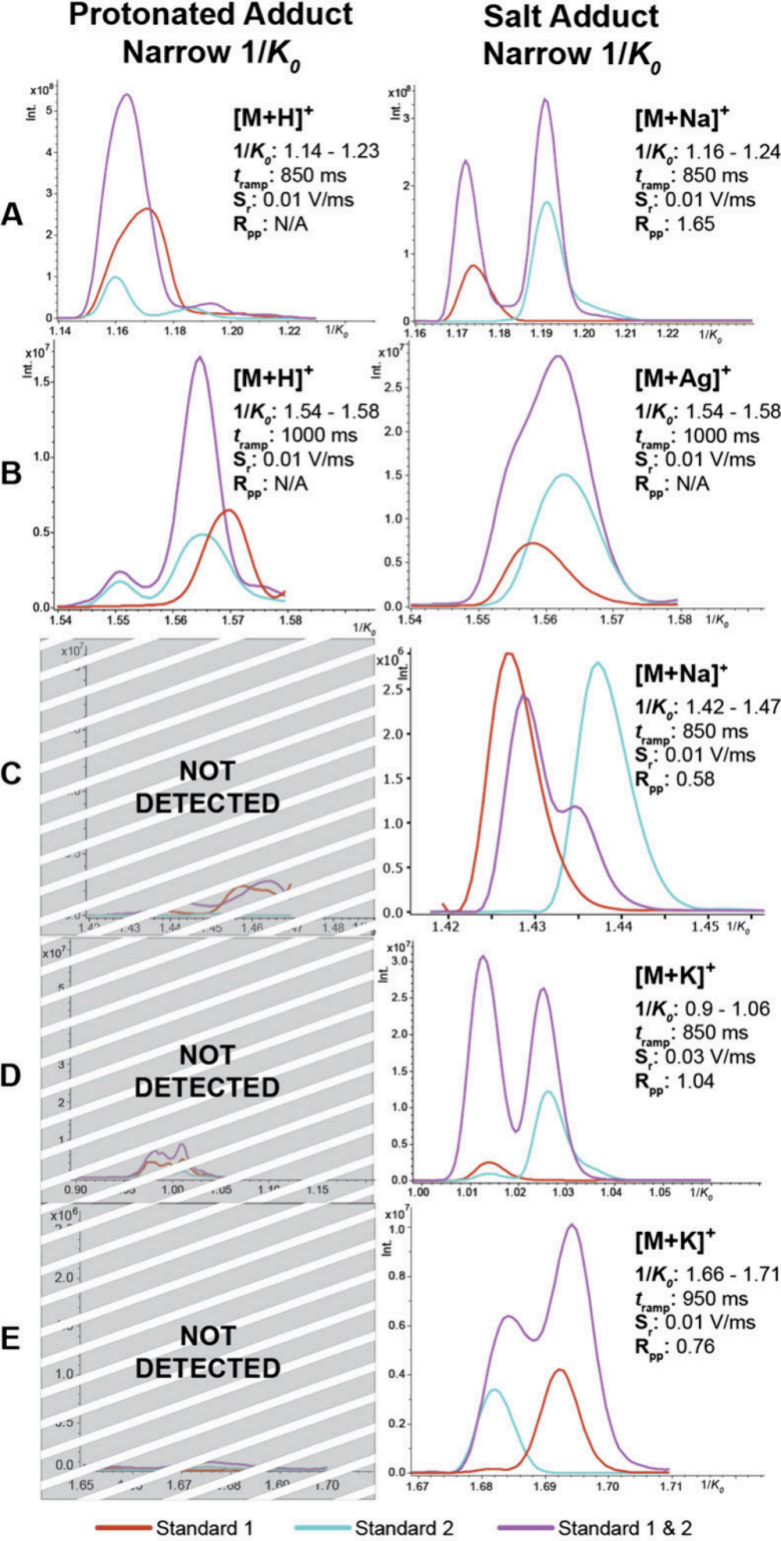
MALDI TIMS IMS of five neutral lipid standards. Red and teal traces correspond to the individually spotted standards, and purple traces represent an isomeric mixture. Standards are displayed as follows: HexCer­(S) (A), HexCer (B), Cer (C), MAG (D), and TAG (E). The salt wash used for each acquisition is indicated by the salt adduct label. Detailed standard information is outlined in Table 1, where Standards 1 and 2 appear sequentially.

With AF, the protonated ions of most standards were not detected or lacked discernible separation using TIMS. Neutral lipid standards exhibited greater sensitivity and specificity upon introducing salt, enabling the separation of most isomers. These findings are consistent with May et al. 2020, demonstrating that cation adduction influences the gas-phase conformation of lipid isomers.^34,42,49^ Thus, salt doping is beneficial for the TIMS-based separation of neutral lipid isomer species. For every isomer pair except the stereoisomer standards (structures depicted in Figure S3b), a high mobility resolving power (e.g., ramp rates of ≤ 0.03 V/ms) was required to achieve at least partial separation. Separation of the isomeric mixture (purple traces depicted in Figure 2) was quantified using two-peak resolution.^42^ While certain isomers, such as the glu- and galactosyl sphingosine standard pair depicted in Figure 2a, can be separated effectively at lower resolving powers (*R*_pp_ = 1.65), other types of stereoisomers, as shown in Figures 2b and S3b, may necessitate more targeted approaches with higher resolving powers for effective separation. One potential avenue to address the separation of stereoisomers is using a higher resolving power ion mobility platform, like cyclic ion mobility.^50^

These observations provided valuable insights into the resolving powers necessary for answering specific biological questions. It is essential to recognize that when utilizing TIMS, there is a trade-off between ion mobility resolving power and inverse mobility (1/*K*_0_) window width. Our data indicated that many stereoisomers required a narrow 1/*K*_0_ range and an extended ramp time to gain sufficient resolving power for isomeric separation. MAG (Figure 2d) and TAG (Figure 2e) isomer pairs demonstrated optimal separation (*R*_pp_ = 1.04, *R*_pp_ = 0.76, respectively) under these high-resolving power conditions. Assessing salt adducts of isomer standards provided predictive insights into isomer separability for subsequent tissue analyses. This method enhances both the sensitivity and specificity of neutral lipid detection, enabling more in-depth MALDI TIMS investigations. While sodiated neutral lipid separations have been achieved using electrospray ionization, these previous methods lack the spatial context necessary for linking molecular observations to distinct histopathological features.^42^

#### High Sensitivity and Specificity Mapping of Neutral Lipids

Salt-enhanced MALDI TIMS IMS was applied to tissue samples for high sensitivity and specificity *in situ* neutral lipid imaging. A human colon tissue section was washed with Na^+^ CBS and imaged at a high spatial resolution (10 μm pixel size). As shown in previous studies, ion mobility dramatically increases the overall molecular coverage for IMS experiments, and this was also true for neutral lipids when introducing salt-doping.^35,51–58^

For example, forty-six TAGs were annotated when washing with Na^+^ CBS, but only five TAGs were annotated on a serial tissue section washed with AF (). The ion mobility heatmap and average mass spectrum depicted in Figure 3a and b highlight the complex molecular profile detected by salt-enhanced MALDI from human colon tissue. Both isobaric and isomeric sodiated neutral lipids were separated using TIMS. For example, several TAGs exhibited an overlap of the [M+] peak of one TAG and the [M + 2] isotopologue peak of another TAG that contains one more double bond (Figure 3c). This double-bond ambiguity is a common isobaric overlap (Δ*m*/*z* 0.00894) that can only be resolved by ultrahigh mass resolution measurements, e.g., using FT-ICR MS with at least 100,000 spectral resolving power at *m*/*z* 900.^59^ This can make annotating lipids difficult using lower-resolution mass analyzers. While HPLC can effectively differentiate between ambiguities of this nature, this is not possible in an imaging context.^60^ MALDI TIMS IMS can, however, distinguish these overlapping species and their spatial localization.

**Figure 3:**
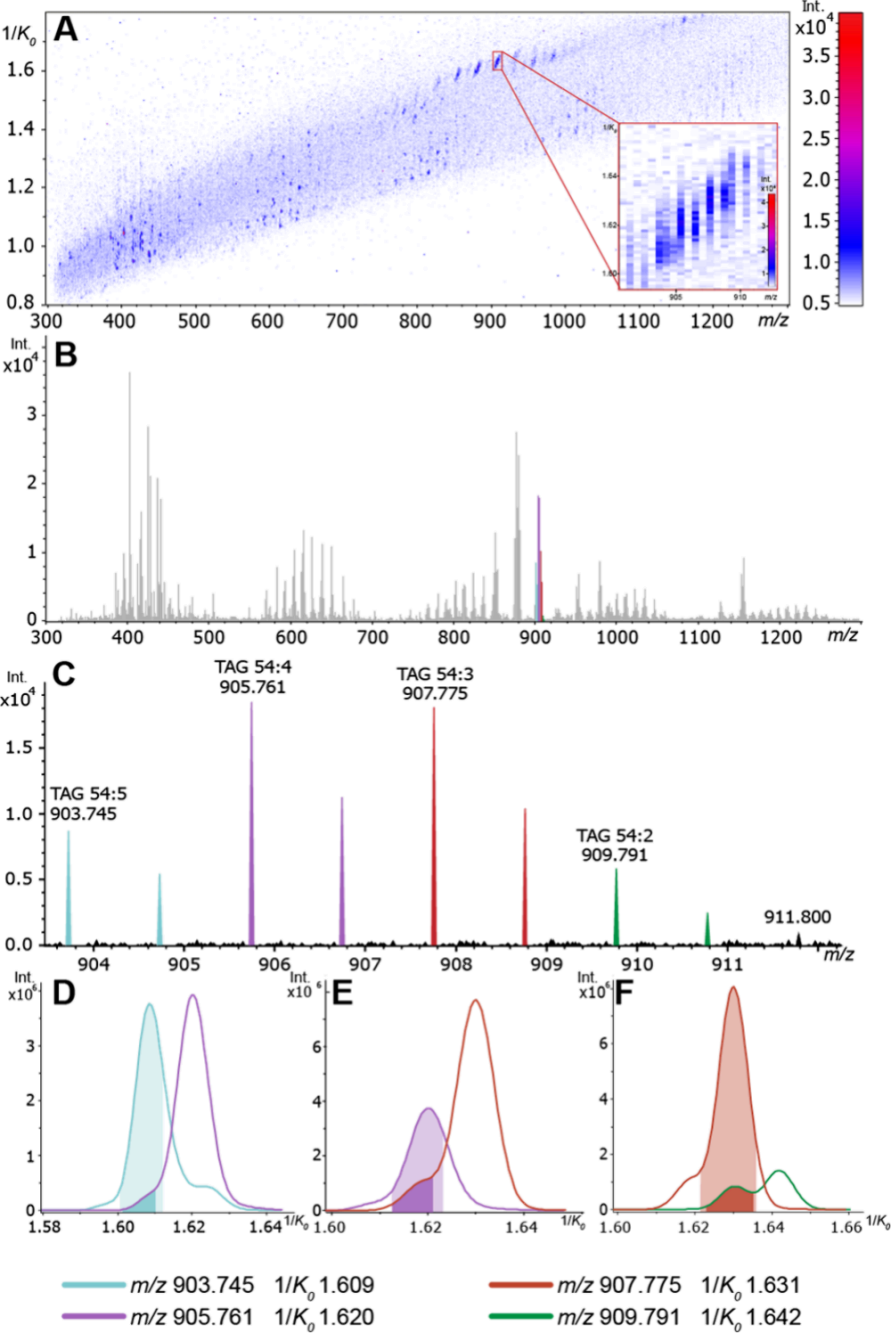
Ion mobility heatmap (A) and averaged mass spectrum (B) collected from MALDI TIMS IMS analysis of a Na^+^ CBS doped human colon tissue section. Isobaric overlaps of four TAG species are depicted in the *m*/*z* dimension (C) and 1/*K*_0_ dimension (D–F). Extracted ion mobility spectra, as shown in D–F, were generated with a ±0.005 Da window. Light-colored shading indicates [M+] peaks and dark shading represents [M + 2] peaks.

Figure 3c shows four TAGs detected by MALDI TIMS IMS, each having one less double-bond with increasing *m*/*z*: [TAG (54:5) + Na]^+^ (*m*/*z* 903.745, 4.20 ppm), [TAG (54:4) + Na]^+^ (*m*/*z* 905.761, 4.53 ppm), [TAG (54:3) + Na]^+^ (*m*/*z* 907.775, 2.75 ppm), and [TAG (54:2) + Na]^+^ (*m*/*z* 909.791, 3.08 ppm). TIMS was able to unravel these overlapping isobaric patterns, revealing the complexity and diversity of neutral lipids in these data (Figure 3d–f).

Isomeric and isobaric lipids from multiple lipid classes were separated with salt-enhanced MALDI TIMS. Figure 4 highlights uniquely distributed DAG isobars and phosphatidylcholine (PC) and TAG isomers in human colon tissue. Figure 4a provides the autofluorescence image of a human colon tissue section that was subsequently doped with Na^+^ CBS. Regions of muscle, mucosa, and submucosa were imaged with TIMS. Isobaric species, DAG 34:3 (*m*/*z* 573.489, 1.39 ppm) and DAG O-32:2 (*m*/*z* 573.486, 2.00 ppm), were found to localize to different areas of the mucosa (Figure 4b).

**Figure 4:**
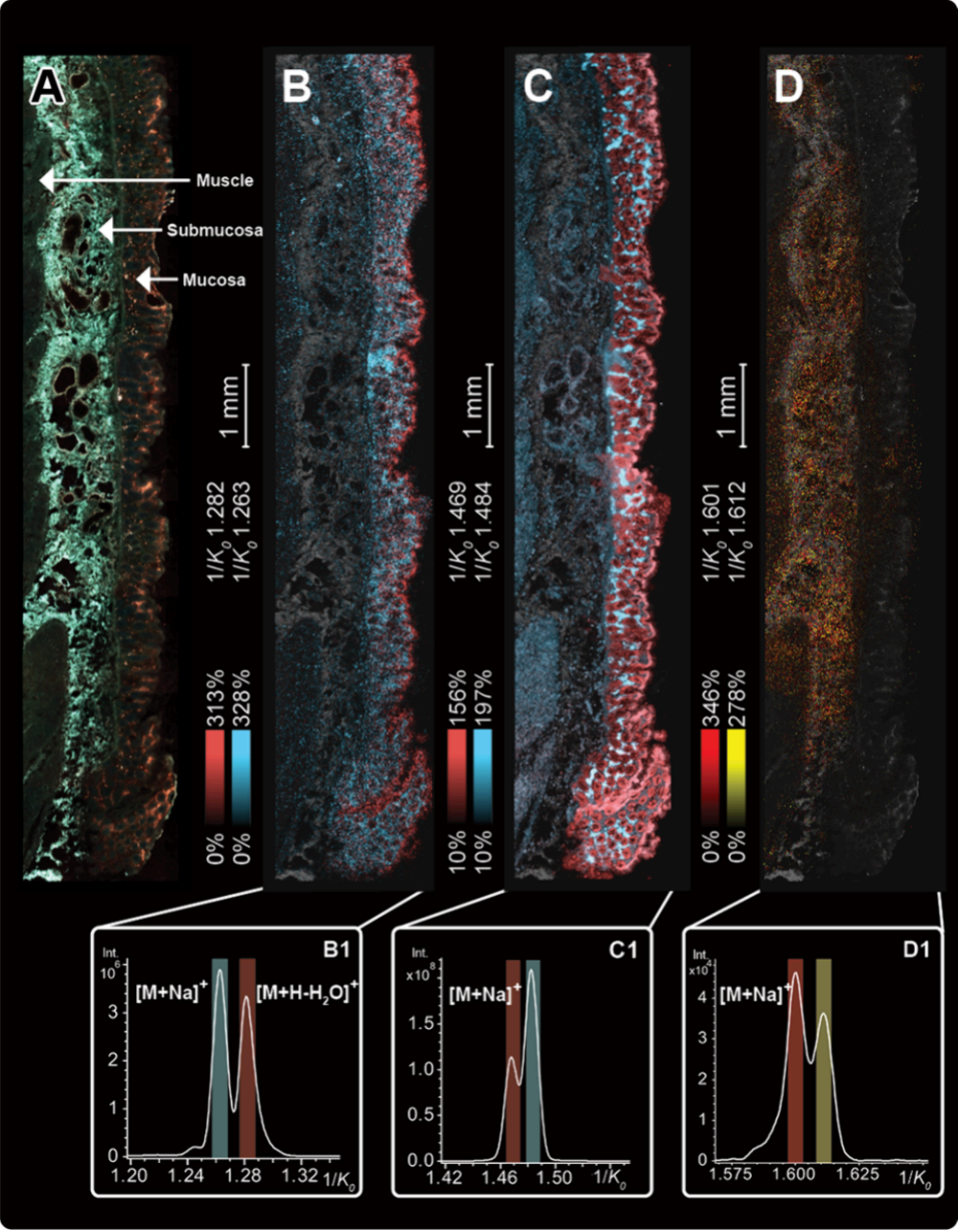
Autofluorescence of a Na^+^ CBS doped human colon tissue section (A) and MALDI TIMS IMS images (B–D) imaged at a 10 μm pixel size in positive mode. Ion images are overlaid on a black-and-white autofluorescence image. The corresponding ion mobility spectra are displayed in (B1–D1).

DAG O-32:2 and DAG 34:3 were differentiated with TIMS (Figure 4b1) and were detected only as the [M + Na]^+^ and [M + H – H_2_O]^+^ adducts, respectively. Furthermore, the Na^+^ CBS wash successfully promoted the sodiation of other isomeric lipid species, such as PC 36:4 (*m*/*z* 804.551, 0.49 ppm) and TAG 50:1 (*m*/*z* 855.743, 2.10 ppm). Figure 4c and d display MALDI TIMS IMS images that show the distributions for two PC 36:4 isomers and TAG 50:1 isomers as [M + Na]^+^ adducts. The corresponding ion mobility spectra are provided in Figure 4c1 and d1. Supporting LC-MS/MS measurements (Figure S4) of PC 36:4 isomers suggest that PC (18:2_18:2) is represented by the peak at 1/*K*_0_ 1.469 and PC (16:0_20:4) has a measured 1/*K*_0_ of 1.484 (Figure 4c1). An average mass spectrum and complementary ultrahigh mass resolution measurements are provided in Figures S5–6, confirming these isobaric and isomeric separations. These results underscore the capability of salt doping for mapping biologically relevant neutral lipid isomer distributions.

## Conclusion

In conclusion, we developed a MALDI TIMS IMS workflow incorporating isotonic metal–cation washes to improve the sensitivity and specificity of neutral lipid detection in a wide variety of biological tissues. This method addresses critical limitations in current MALDI IMS workflows, including the low ionization efficiency and structural ambiguity inherent to neutral lipids. Although all salt washes tested showed improved neutral lipid detection for most molecular classes, Na^+^ CBS demonstrated the most consistent performance enhancement across the broadest range of neutral lipid classes for the widest variety of tissue types. Salt-enhanced MALDI IMS also has the advantage of simplifying the spectral complexity for positive ion mode imaging experiments by driving the majority of ions to the same adduct, improving lipid annotation accuracy and analytical sensitivity. Moreover, integrating TIMS provided critical orthogonal separation, effectively resolving neutral lipid isomers and isobars, which were previously indistinguishable by traditional IMS methods alone. Metal cationization dramatically improved the ability of TIMS to resolve structural differences in neutral lipids compared with protonated ions. The combined salt-enhanced TIMS workflow enabled detailed spatial mapping and differentiation of neutral lipid isomers within histologically relevant tissue structures, notably identifying distinct spatial distributions of DAGs and TAGs in human colon tissue. This approach is particularly advantageous over other methodologies because it is easy to implement into typical sample preparation workflows, is cost-effective, and is compatible with subsequent multimodal analyses, thus expanding its utility in integrative spatial biology studies. However, the inherent complexity of TIMS IMS data sets still poses a challenge when distinguishing and annotating isomers and isobars. Additional work to develop improved computational methods is needed to perform these analyses at scale. Still, the capabilities of salt-enhanced MALDI TIMS substantially advance the potential for IMS research in understanding the role of neutral lipids in complex cellular processes and disease states.

## Supplementary Material





## Notes

LC-MS/MS methods, average mass spectra, comparison of neutral lipid annotations by tissue, MALD TIMS IMS of neutral lipid isomer standards with wide 1/*K*_0_ and structural information, and validatory LC-MS/MS chromatograms (PDF)
Experimental parameters and all neutral lipid annotations (XLSX)

## References

[1] Conroy M., Andrews R., Andrews S., Cockayne L., Dennis E., Fahy E., Gaud C., Griffiths W., Jukes G., Kolchin M., Mendivelso K., Lopez-Clavijo A., Ready C., Subramaniam S., O’Donnell V. (2024). LIPID MAPS: Update to databases and tools for the lipidomics
community. Nucleic Acids Research.

[2] Olzmann J. A., Carvalho P. (2019). Dynamics and Functions of Lipid Droplets. Nat. Rev. Mol. Cell Biol..

[3] Chew H., Solomon V. A., Fonteh A. N. (2020). Involvement
of Lipids in Alzheimer’s
Disease Pathology and Potential Therapies. Front.
Physiol..

[4] Kao Y.-C., Ho P.-C., Tu Y.-K., Jou I.-M., Tsai K.-J. (2020). Lipids
and Alzheimer’s Disease. IJMS.

[5] Maxfield F. R., Tabas I. (2005). Role of Cholesterol
and Lipid Organization in Disease. Nature.

[6] Shamim A., Mahmood T., Ahsan F., Kumar A., Bagga P. (2018). Lipids: An
Insight into the Neurodegenerative Disorders. Clinical Nutrition Experimental.

[7] Akyol S., Ugur Z., Yilmaz A., Ustun I., Gorti S. K. K., Oh K., McGuinness B., Passmore P., Kehoe P. G., Maddens M. E., Green B. D., Graham S. F. (2021). Lipid Profiling
of Alzheimer’s Disease Brain Highlights Enrichment in Glycerol­(Phospho)­Lipid,
and Sphingolipid Metabolism. Cells.

[8] Colley M. E., Esselman A. B., Scott C. F., Spraggins J. M. (2024). High-Specificity
Imaging Mass Spectrometry. Annual Review of
Analytical Chemistry.

[9] Yang K., Dilthey B. G., Gross R. W. (2016). Shotgun
Lipidomics Approach to Stabilize
the Regiospecificity of Monoglycerides Using a Facile Low-Temperature
Derivatization Enabling Their Definitive Identification and Quantitation. Anal. Chem..

[10] Holbrook J. H., Kemper G. E., Hummon A. B. (2024). Quantitative
Mass Spectrometry Imaging:
Therapeutics & Biomolecules. Chem. Commun..

[11] Zemski
Berry K. A., Hankin J. A., Barkley R. M., Spraggins J. M., Caprioli R. M., Murphy R. C. (2011). MALDI Imaging of Lipid Biochemistry
in Tissues by Mass Spectrometry. Chem. Rev..

[12] Li D., Ouyang Z., Ma X. (2023). Mass Spectrometry
Imaging for Single-Cell
or Subcellular Lipidomics: A Review of Recent Advancements and Future
Development. Molecules.

[13] Bowman A. P., Blakney G. T., Hendrickson C. L., Ellis S. R., Heeren R. M. A., Smith D. F. (2020). Ultra-High Mass
Resolving Power, Mass Accuracy, and
Dynamic Range MALDI Mass Spectrometry Imaging by 21-T FT-ICR MS. Anal. Chem..

[14] Mast D. H., Liao H.-W., Romanova E. V., Sweedler J. V. (2021). Analysis of Peptide
Stereochemistry in Single Cells by Capillary Electrophoresis-Trapped
Ion Mobility Spectrometry Mass Spectrometry. Anal. Chem..

[15] Zahraei A., Guo G., Perwick R. D., Donaldson P. J., Demarais N. J., Grey A. C. (2021). Mapping
Glucose Metabolites in the Normal Bovine Lens: Evaluation and Optimisation
of a Matrix-assisted Laser Desorption/Ionisation Imaging Mass Spectrometry
Method. J. Mass Spectrom.

[16] Kruse A. R. S., Judd A. M., Gutierrez D. B., Allen J. L., Dufresne M., Farrow M. A., Powers A. C., Norris J. L., Caprioli R. M., Spraggins J. M. (2024). Thermal
Denaturation of Fresh Frozen Tissue Enhances
Mass Spectrometry Detection of Peptides. Anal.
Chem..

[17] Groseclose M. R., Andersson M., Hardesty W. M., Caprioli R. M. (2007). Identification
of
Proteins Directly from Tissue: In Situ Tryptic Digestions Coupled
with Imaging Mass Spectrometry. J. Mass Spectrom.

[18] Sparvero L.
J., Amoscato A. A., Dixon C. E., Long J. B., Kochanek P. M., Pitt B. R., Bayır H., Kagan V. E. (2012). Mapping of Phospholipids
by MALDI Imaging (MALDI-MSI): Realities and Expectations. Chem. Phys. Lipids.

[19] Jones E. E., Dworski S., Canals D., Casas J., Fabrias G., Schoenling D., Levade T., Denlinger C., Hannun Y. A., Medin J. A., Drake R. R. (2014). On-Tissue Localization
of Ceramides and Other Sphingolipids by MALDI Mass Spectrometry Imaging. Anal. Chem..

[20] Xu L., Kliman M., Forsythe J. G., Korade Z., Hmelo A. B., Porter N. A., McLean J. A. (2015). Profiling
and Imaging Ion Mobility-Mass
Spectrometry Analysis of Cholesterol and 7-Dehydrocholesterol in Cells
Via Sputtered Silver MALDI. J. Am. Soc. Mass
Spectrom..

[21] Fincher J. A., Djambazova K. V., Klein D. R., Dufresne M., Migas L. G., Van de Plas R., Caprioli R. M., Spraggins J. M. (2021). Molecular
Mapping of Neutral Lipids Using Silicon Nanopost Arrays and TIMS Imaging
Mass Spectrometry. J. Am. Soc. Mass Spectrom..

[22] Unsihuay D., Qiu J., Swaroop S., Nagornov K. O., Kozhinov A. N., Tsybin Y. O., Kuang S., Laskin J. (2020). Imaging of Triglycerides in Tissues
Using Nanospray Desorption Electrospray Ionization (Nano-DESI) Mass
Spectrometry. Int. J. Mass Spectrom..

[23] Sugiura Y., Setou M. (2009). Selective Imaging of
Positively Charged Polar and Nonpolar Lipids
by Optimizing Matrix Solution Composition. Rapid
Commun. Mass Spectrom..

[24] Hsu F.-F., Turk J. (1999). Structural Characterization
of Triacylglycerols as Lithiated Adducts
by Electrospray Ionization Mass Spectrometry Using Low-Energy Collisionally
Activated Dissociation on a Triple Stage Quadrupole Instrument. J. Am. Soc. Mass Spectrom..

[25] Lau W. C. D., Donnellan L., Briggs M., Rupasinghe T., Harris J. C., Hayes J. E., Hoffmann P. (2024). Sodium Doping and Trapped
Ion Mobility Spectrometry Improve Lipid Detection for Novel MALDI-MSI
Analysis of Oats. Food Chem..

[26] Dufresne M., Masson J.-F., Chaurand P. (2016). Sodium-Doped
Gold-Assisted Laser
Desorption Ionization for Enhanced Imaging Mass Spectrometry of Triacylglycerols
from Thin Tissue Sections. Anal. Chem..

[27] Dufresne M., Patterson N. H., Norris J. L., Caprioli R. M. (2019). Combining Salt Doping
and Matrix Sublimation for High Spatial Resolution MALDI Imaging Mass
Spectrometry of Neutral Lipids. Anal. Chem..

[28] Picariello G., Paduano A., Sacchi R., Addeo F. (2009). MALDI-TOF Mass Spectrometry
Profiling of Polar and Nonpolar Fractions in Heated Vegetable Oils. J. Agric. Food Chem..

[29] Pittenauer E., Allmaier G. (2009). The Renaissance of
High-Energy CID for Structural Elucidation
of Complex Lipids: MALDI-TOF/RTOF-MS of Alkali Cationized Triacylglycerols. J. Am. Soc. Mass Spectrom..

[30] McMillen J. C., Fincher J. A., Klein D. R., Spraggins J. M., Caprioli R. M. (2020). Effect of MALDI Matrices on Lipid
Analyses of Biological
Tissues Using MALDI-2 Postionization Mass Spectrometry. J. Mass Spectrom.

[31] Soltwisch J., Kettling H., Vens-Cappell S., Wiegelmann M., Müthing J., Dreisewerd K. (2015). Mass Spectrometry
Imaging with Laser-Induced
Postionization. Science.

[32] Habler K., Rexhaj A., Adling-Ehrhardt M., Vogeser M. (2024). Understanding Isotopes,
Isomers, and Isobars in Mass Spectrometry. Journal
of Mass Spectrometry and Advances in the Clinical Lab.

[33] Smith D. F., Kiss A., Leach F. E., Robinson E. W., Paša-Tolić L., Heeren R. M. A. (2013). High
Mass Accuracy and High Mass Resolving Power FT-ICR
Secondary Ion Mass Spectrometry for Biological Tissue Imaging. Anal Bioanal Chem..

[34] Camunas-Alberca S. M., Moran-Garrido M., Sáiz J., Gil-de-la-Fuente A., Barbas C., Gradillas A. (2023). Integrating
the Potential of Ion
Mobility Spectrometry-Mass Spectrometry in the Separation and Structural
Characterisation of Lipid Isomers. Front. Mol.
Biosci..

[35] Djambazova K. V., Klein D. R., Migas L. G., Neumann E. K., Rivera E. S., Van de Plas R., Caprioli R. M., Spraggins J. M. (2020). Resolving
the Complexity of Spatial Lipidomics Using MALDI TIMS Imaging Mass
Spectrometry. Anal. Chem..

[36] Clowers B. H., Dwivedi P., Steiner W. E., Hill H. H., Bendiak B. (2005). Separation
of Sodiated Isobaric Disaccharides and Trisaccharides Using Electrospray
Ionization-Atmospheric Pressure Ion Mobility-Time of Flight Mass Spectrometry. J. Am. Soc. Mass Spectrom..

[37] Groessl M., Graf S., Knochenmuss R. (2015). High Resolution
Ion Mobility-Mass
Spectrometry for Separation and Identification of Isomeric Lipids. Analyst.

[38] Angel P. M., Spraggins J. M., Baldwin H. S., Caprioli R. (2012). Enhanced Sensitivity
for High Spatial Resolution Lipid Analysis by Negative Ion Mode Matrix
Assisted Laser Desorption Ionization Imaging Mass Spectrometry. Anal. Chem..

[39] Dufresne M., Migas L., Djambazova K., Colley M., Van De Plas R., Spraggins J. (2025). Aminated Cinnamic
Acid Analogs as Dual Polarity Matrices
for High Spatial Resolution MALDI Imaging Mass Spectrometry. ChemRxiv.

[40] Neumann E., Allen J., Brewer M., Anderson D., De Caestecker M., Gutierrez D., Spraggins J. (2020). VU Biomolecular Multimodal Imaging
Center (BIOMIC) Kidney Characterization Pipeline for Tissues Collected
through the Cooperative Human Tissue Network (CHTN) V4. Protocols.io.

[41] Dodds J. N., May J. C., McLean J. A. (2017). Investigation of
the Complete Suite
of the Leucine and Isoleucine Isomers: Toward Prediction of Ion Mobility
Separation Capabilities. Anal. Chem..

[42] May J. C., Knochenmuss R., Fjeldsted J. C., McLean J. A. (2020). Resolution of Isomeric
Mixtures in Ion Mobility Using a Combined Demultiplexing and Peak
Deconvolution Technique. Anal. Chem..

[43] Migas L. (2023). IMS Data Processing
V1. Protocols.io.

[44] Migas L. (2023). Untargeted
IMS Tentative Identification Lipidomics V1. Protocols.io.

[45] Sud M., Fahy E., Cotter D., Brown A., Dennis E. A., Glass C. K., Merrill A. H., Murphy R. C., Raetz C. R. H., Russell D. W., Subramaniam S. (2007). LMSD: LIPID MAPS Structure Database. Nucleic Acids Res..

[46] Al-Saad K. A., Zabrouskov V., Siems W. F., Knowles N. R., Hannan R. M., Hill H. H. (2003). Matrix-assisted Laser Desorption/Ionization Time-of-flight
Mass Spectrometry of Lipids: Ionization and Prompt Fragmentation Patterns. Rapid Commun. Mass Spectrom..

[47] Gidden J., Liyanage R., Durham B., Lay J. O. (2007). Reducing Fragmentation
Observed in the Matrix-assisted Laser Desorption/Ionization Time-of-flight
Mass Spectrometric Analysis of Triacylglycerols in Vegetable Oils. Rapid Commun. Mass Spectrom..

[48] Wang H.-Y. J., Liu C. B., Wu H.-W. (2011). A Simple
Desalting Method for Direct
MALDI Mass Spectrometry Profiling of Tissue Lipids. J. Lipid Res..

[49] Jeanne
Dit Fouque K., Ramirez C. E., Lewis R. L., Koelmel J. P., Garrett T. J., Yost R. A., Fernandez-Lima F. (2019). Effective
Liquid Chromatography-Trapped Ion Mobility Spectrometry-Mass Spectrometry
Separation of Isomeric Lipid Species. Anal.
Chem..

[50] Leontyev D., Olivos H., Shrestha B., Datta Roy P. M., LaPlaca M. C., Fernández F. M. (2024). Desorption Electrospray Ionization
Cyclic Ion Mobility-Mass Spectrometry Imaging for Traumatic Brain
Injury Spatial Metabolomics. Anal. Chem..

[51] McLean J. A., Ridenour W. B., Caprioli R. M. (2007). Profiling and Imaging of Tissues
by Imaging Ion Mobility-mass Spectrometry. J.
Mass Spectrom..

[52] Zhang H., Liu Y., Fields L., Shi X., Huang P., Lu H., Schneider A. J., Tang X., Puglielli L., Welham N. V., Li L. (2023). Single-Cell
Lipidomics Enabled by
Dual-Polarity Ionization and Ion Mobility-Mass Spectrometry Imaging. Nat. Commun..

[53] Ellis, S. R. ; Soltwisch, J. Combining Ion Mobility of Lipids with MSI. In Mass Spectrometry for Lipidomics; Holčapek, M. , Ekroos, K. , Eds.; Wiley, 2023; pp 128–129. 10.1002/9783527836512.ch5.

[54] Spraggins J. M., Djambazova K. V., Rivera E. S., Migas L. G., Neumann E. K., Fuetterer A., Suetering J., Goedecke N., Ly A., Van de Plas R., Caprioli R. M. (2019). High-Performance Molecular Imaging
with MALDI Trapped Ion-Mobility Time-of-Flight (timsTOF) Mass Spectrometry. Anal. Chem..

[55] Rivera E. S., Djambazova K. V., Neumann E. K., Caprioli R. M., Spraggins J. M. (2020). Integrating
Ion Mobility and Imaging Mass Spectrometry for Comprehensive Analysis
of Biological Tissues: A Brief Review and Perspective. J. Mass Spectrom.

[56] Djambazova K. V., Dufresne M., Migas L. G., Kruse A. R. S., Van
de Plas R., Caprioli R. M., Spraggins J. M. (2023). MALDI TIMS
IMS of Disialoganglioside Isomers—GD1a and GD1b in Murine Brain
Tissue. Anal. Chem..

[57] Helmer P. O., Nordhorn I. D., Korf A., Behrens A., Buchholz R., Zubeil F., Karst U., Hayen H. (2021). Complementing Matrix-Assisted
Laser Desorption Ionization-Mass Spectrometry Imaging with Chromatography
Data for Improved Assignment of Isobaric and Isomeric Phospholipids
Utilizing Trapped Ion Mobility-Mass Spectrometry. Anal. Chem..

[58] Hale, O. J. ; Illes-Toth, E. ; Sisley, E. K. ; Cooper, H. J. Ion Mobility Spectrometry in Mass Spectrometry Imaging. In New Developments in Mass Spectrometry; Ashcroft, A. E. , Sobott, F. , Eds.; Royal Society of Chemistry: Cambridge, 2021; pp 272–306. 10.1039/9781839162886-00272.

[59] Bielow C., Mastrobuoni G., Orioli M., Kempa S. (2017). On Mass Ambiguities
in High-Resolution Shotgun Lipidomics. Anal.
Chem..

[60] Fauland A., Köfeler H., Trötzmüller M., Knopf A., Hartler J., Eberl A., Chitraju C., Lankmayr E., Spener F. (2011). A Comprehensive Method for Lipid
Profiling by Liquid
Chromatography-Ion Cyclotron Resonance Mass Spectrometry. J. Lipid Res..

