# Supplementary material for: High-Specificity and Sensitivity Imaging of Neutral Lipids Using Salt-Enhanced MALDI TIMS
Source: J Am Soc Mass Spectrom. 2025 Sep 4;36(10):2213–21. doi: 10.1021/jasms.5c00202 (PMC12492385; doi:10.1021/jasms.5c00202)
Supplement: Supplementary file 1 [file js5c00202_si_001.pdf]

Supporting Information

High-Specificity and Sensitivity Imaging of Neutral Lipids using Salt-Enhanced MALDI TIMS

Kameron R. Molloy[1,2], Martin Dufresne[2,3], Madeline E. Colley[2,3], Lukasz G. Migas[2,4], Raf Van de Plas[2,4,5], Jeffrey M. Spraggins[1,2,3,5,6*]

[1]Department of Chemistry, Vanderbilt University, Nashville, TN 37235

[2]Mass Spectrometry Research Center, Vanderbilt University, Nashville, TN 37235

[3]Department of Cell and Developmental Biology, Vanderbilt University, Nashville, TN 37232

[4]Delft Center for Systems and Control, Delft University of Technology, 2628 Delft, Netherlands

[5]Department of Biochemistry, Vanderbilt University, Nashville, TN 37205

[6]Department of Pathology, Microbiology and Immunology, Vanderbilt University Medical Center, Nashville, TN 37205

*Corresponding author: Email – jeff.spraggins@vanderbilt.edu

SUPPORTING INFORMATION

SECTION S1

LC-MS/MS by 4D-PASEF Methods

In parallel to MALDI TIMS IMS experiments, LC-MS/MS was performed to confirm lipid annotations for all tissues. For each tissue (rabbit adrenal gland, murine brain, human kidney, and human colon), 10 µm thick fresh frozen sections were collected in glass vials using a Leica CM3050 cryostat (Leica Microsystems GmbH, Wetzlar, Germany). Each glass vial consisted of three sections from a single organ tissue, 5 µL Avanti Equisplash mixture, 5 µL C24:1 mono-sulfo galactosyl(ß) ceramide-d7 (d18:1/24:1) (Avanti), and 5 µL 18:2 Cardiolipin-d5 (Avanti). All spiked standards had a concentration of 100 µg/mL. Next, metal beads were placed in the vials then methanol was added to totally submerge the tissue. The mixture was vortexed for 1 minute, let to sit over dry ice for 5 minutes, then sonicated over ice for 30 minutes. Lipid extraction was performed by adding 1600 µL of MTBE and 400 µL of methanol (both at 4°C). Finally, the solutions were centrifuged at 4°C for 10 minutes at 100 × *g* before allowing to rest over ice for 10 minutes. The top layer was transferred to a new vial and then dried down with nitrogen before being resuspended in 300 µL of methanol. A timsTOF Pro2 mass spectrometer coupled to a Waters Premier QSM was utilized for positive and negative ion mode 4D-PASEF LC-MS/MS analysis of lipid extracts from each tissue type. A 100 mm x 2.1 mm Waters Premier CSH-C18 column was used for separation where solvent A) 40:60 H2O:ACN with 10 mM ammonium formate and 0.1% formic acid and B) 90:10 IPA:ACN with 10 mM ammonium formate and 0.1% formic acid. Positive mode as collected using the Bruker default 4D-PASEF method and negative mode was collected using an in-house developed 4D-PASEF method with MS/MS stepping. Resulting data was annotated using MS-DIAL 4.9 and only reference matched species were considered for library generation.

TABLE S1. Molarities of carbonate buffer solution components.

Reagent	Concentration (Stock)	Concentration (Final - after 2.5x dilution)
Sodium Carbonate	29.32 mM	11.73 mM
Sodium Bicarbonate	70.68 mM	28.27 mM
Sodium Acetate	249.89 mM	99.96 mM

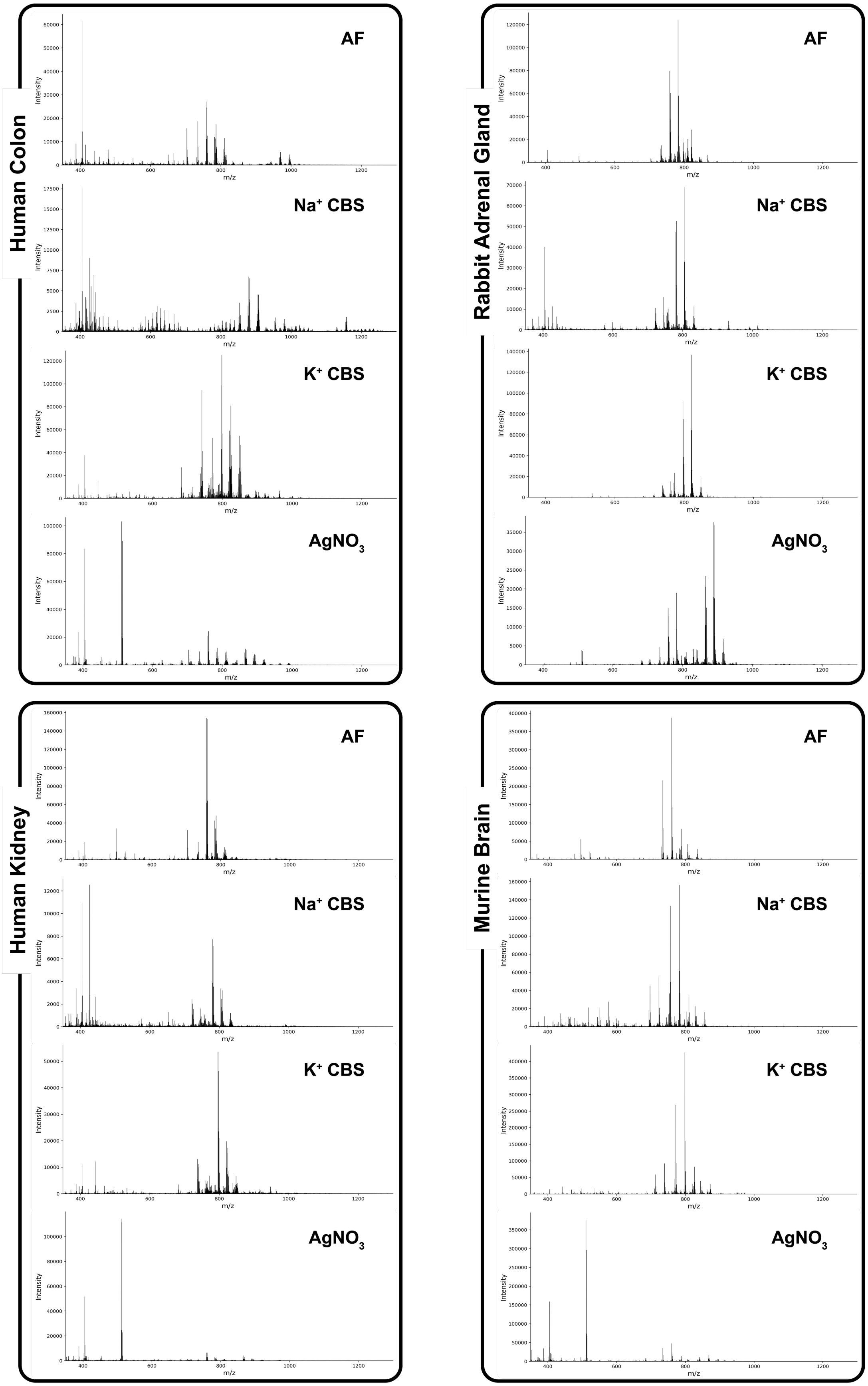

Figure S1. MALDI TIMS IMS average mass spectra collected for each tissue with every salt wash. Salt washes are indicated in the top right of every spectrum.

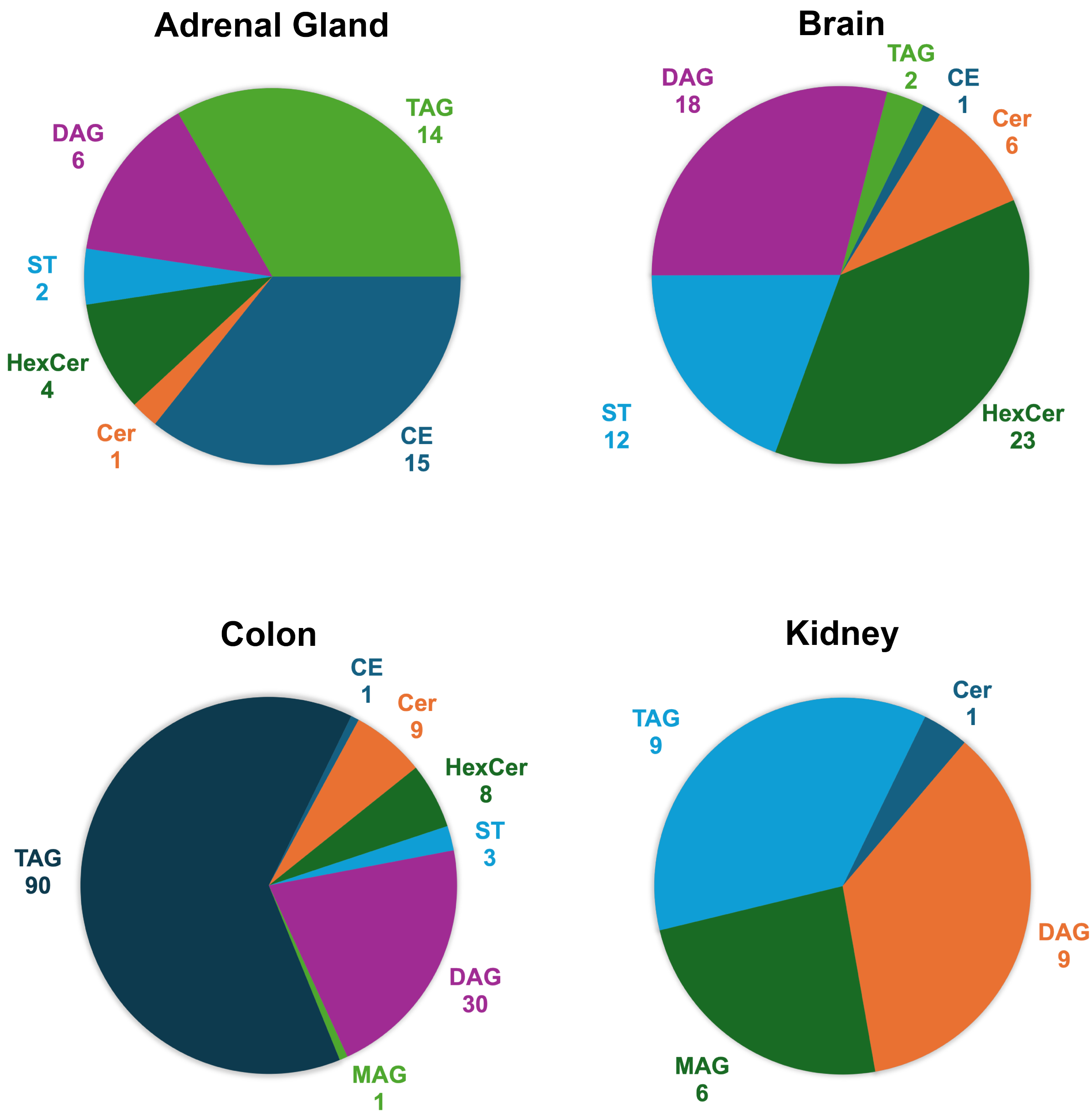

Figure S2. Number of annotations in each neutral lipid subclass that were detected by both LC-MS/MS and MALDI.

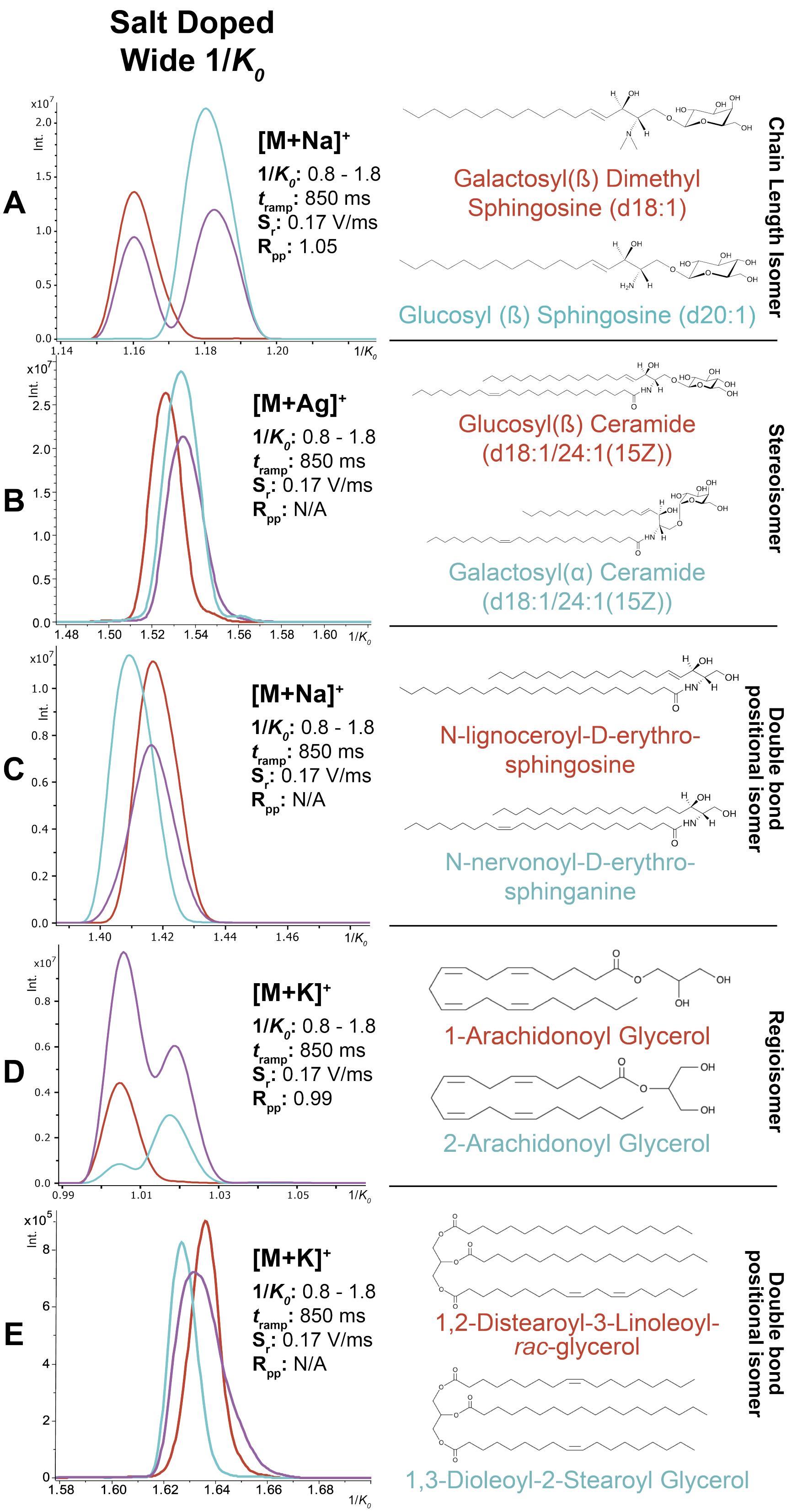

Figure S3. MALDI TIMS IMS of five neutral lipid standards. Red and teal traces correspond to the structure labels in red and teal to the right. Purple traces represent the data acquired when a mix of the isomers was evaluated. Standards are displayed as follows: A: HexCer(S), B: HexCer, C: Cer, D: MAG, E: TAG. The salt wash used for each acquisition is indicated by the salt adduct label. Detailed standard information outlined in Table 1.

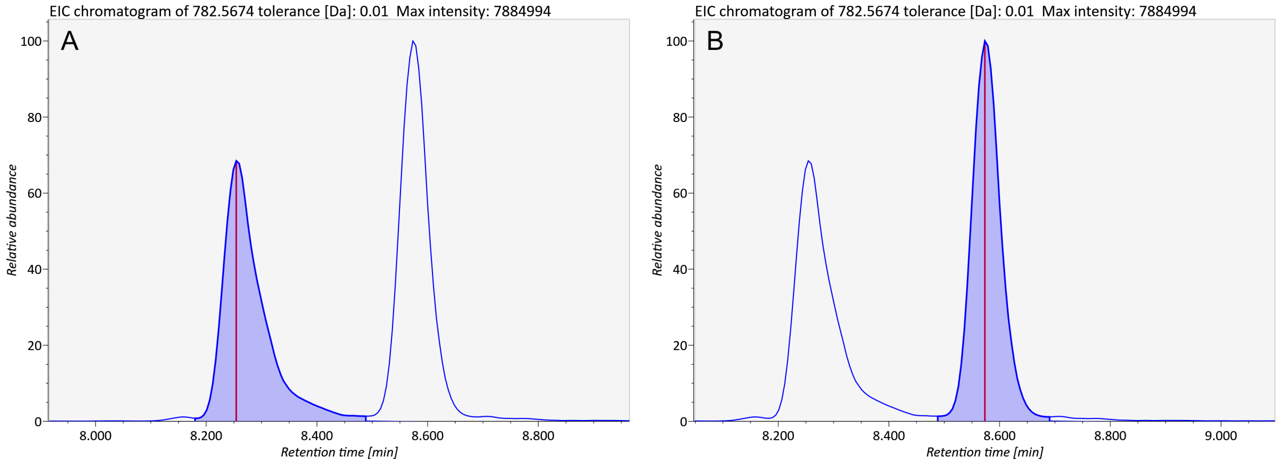

Figure S4. LC-MS/MS chromatogram of PC (36:4) isomers detected as the $[M+H]^+$ adduct. **A.** Chromatographic peak of PC (18:2_18:2) shaded in blue. **B.** Chromatographic peak of PC (16:0_20:4) shaded in blue. Extracted ion chromatograms (EIC) were generated using MS-DIAL 4.9.

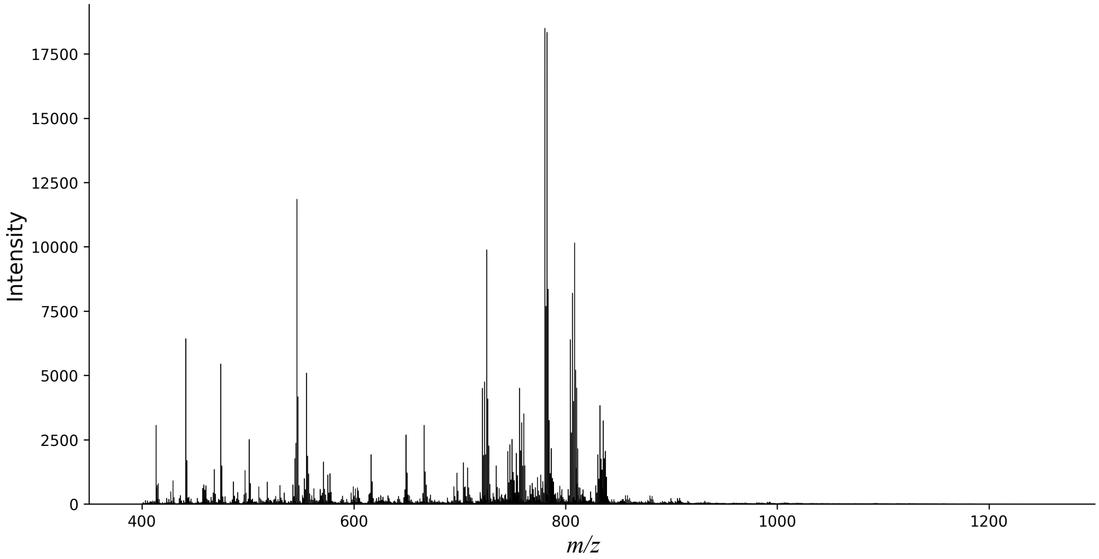

Figure S5. Average mass spectrum of Na^+ CBS doped human colon collected using MALDI TIMS IMS on a Bruker timsTOF fleX.

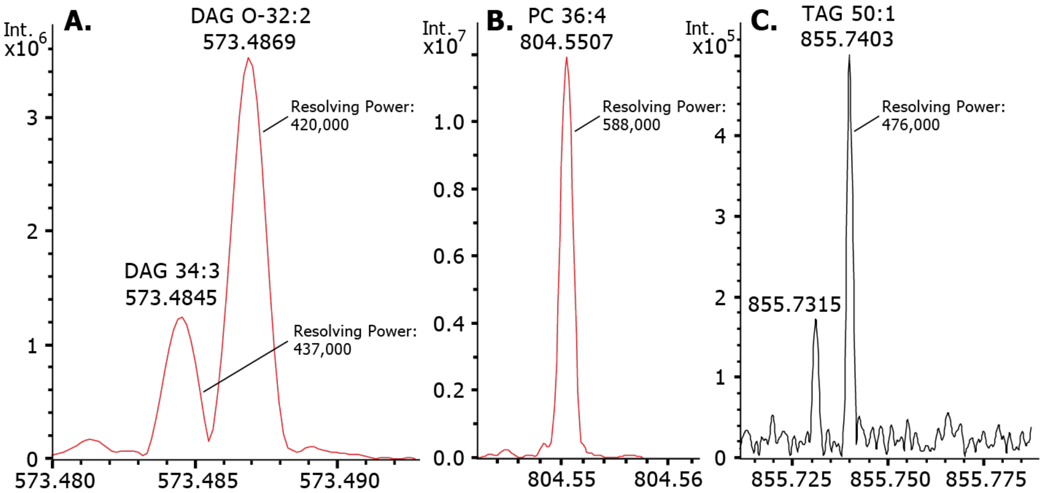

Figure S6. MALDI mass spectra collected of Na^+ CBS doped human colon tissue on a Bruker SolariX 15T FT-ICR. This data validated the identification of DAG isobars (A), PC isomers (B), and TAG isomers (C) reported in Fig. 4.
